# Robot-Assisted Ileocecal Resection for Cecal Cancer with a Common Trunk of the Middle Colic and Ileocolic Arteries: A Case Report

**DOI:** 10.70352/scrj.cr.26-0059

**Published:** 2026-04-09

**Authors:** Yasuki Akiyama, Kei Nakada, Hiroto Sannomiya, Kensuke Nitta, Atsuhiro Koga, Yasuhisa Mori, Masumi Yamauchi, Jun Nagata, Nagahiro Sato, Toshihisa Tamura, Kazunori Shibao, Keiji Hirata

**Affiliations:** Department of Surgery1, University of Occupational and Environmental Health, Kitakyushu, Fukuoka, Japan

**Keywords:** cecal cancer, vascular variation, robot-assisted surgery, ileocecal resection

## Abstract

**INTRODUCTION:**

Vascular variations around the superior mesenteric vessels increase the technical difficulty and risk of injury during right-sided colon surgery. We report a rare case of cecal cancer with a common arterial trunk formed by the middle colic artery (MCA) and ileocolic artery (ICA) treated by robot-assisted surgery.

**CASE PRESENTATION:**

A man in his 70s was diagnosed with cecal cancer during evaluation for anemia. Preoperative CT revealed that the MCA and ICA originated from a common arterial trunk. Robot-assisted ileocecal resection with lymphadenectomy was performed. After identification of the common trunk, only the ICA was divided following confirmation of the bifurcation. Intracorporeal overlap anastomosis was completed using the double bipolar method.

**CONCLUSIONS:**

Robot-assisted surgery with precise preoperative imaging enables safe management of rare mesenteric vascular variations and provides important educational value.

## INTRODUCTION

Anatomical variations of mesenteric vessels are common and can significantly increase the technical difficulty of right-sided colon surgery. In particular, variations involving the middle colic artery (MCA) and ileocolic artery (ICA) may pose a risk of inadvertent vascular injury during lymphadenectomy around the superior mesenteric vessels.^1–4)^ With the increasing use of minimally invasive approaches, including robot-assisted surgery, precise anatomical understanding and safe dissection techniques have become increasingly important.

We report a rare case of cecal cancer in which the MCA and ICA formed a common arterial trunk, successfully managed by robot-assisted ileocecal resection using the double bipolar method.

## CASE PRESENTATION

**Fig. 1:**
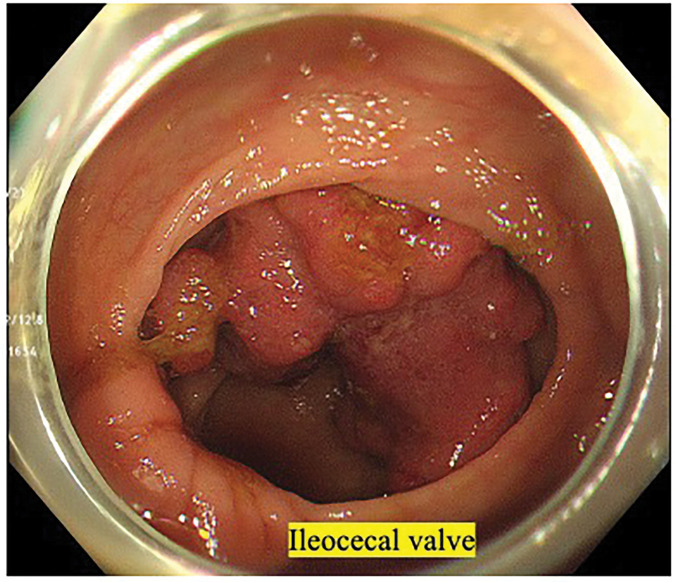
Endoscopic findings. Endoscopic findings showing a tumor located in the cecum near the ileocecal valve.

A man in his 70s was referred to our department after being diagnosed with cecal cancer during evaluation for anemia. Colonoscopy revealed a tumor in the cecum, and biopsy confirmed well-differentiated adenocarcinoma (**Fig. 1**).

**Fig. 2:**
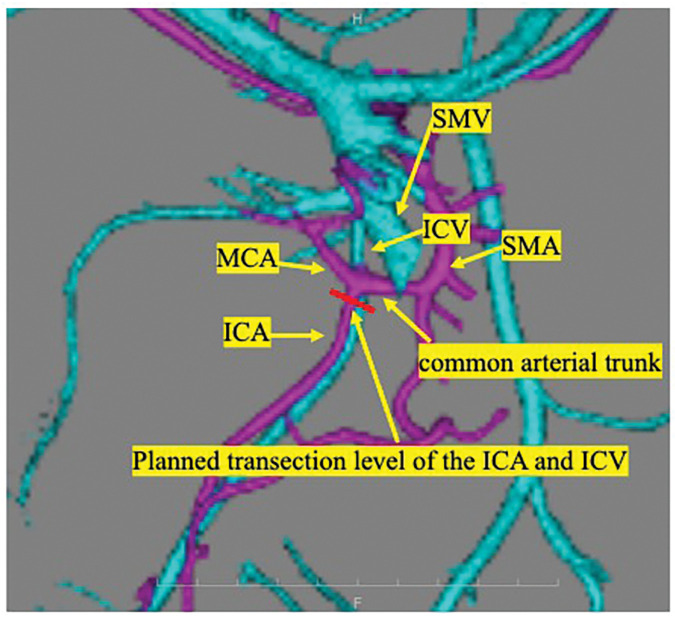
Preoperative CTA. Preoperative CTA demonstrating a common arterial trunk formed by the MCA and ICA. The ICV runs cranially to the common arterial trunk. The planned transection level of the ICA and ICV at the same level is indicated. ICA, ileocolic artery; ICV, ileocolic vein; MCA, middle colic artery; SMA, superior mesenteric artery; SMV, superior mesenteric vein

Contrast-enhanced CT demonstrated no evidence of lymph node or distant metastases. However, a vascular anomaly was identified: the MCA and ICA originated from a single common arterial trunk branching from the superior mesenteric artery (**Fig. 2**). This trunk coursed dorsal to the superior mesenteric vein (SMV). Based on these findings, robot-assisted ileocecal resection with D3 lymphadenectomy (extended lymph node dissection involving central nodes along the SMV) was planned.

### Surgical technique

Robot-assisted ileocecal resection was performed with the patient in the semi-lithotomy position. After lateral mobilization, the SMV was exposed. A common arterial trunk running dorsal to the SMV was identified, and the ileocolic vein was confirmed cranial to the common arterial trunk.

**Fig. 3:**
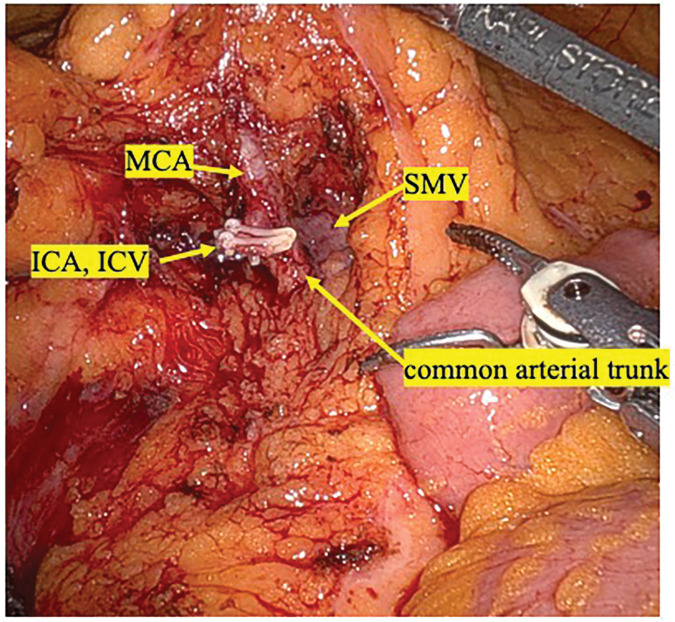
After completion of lymphadenectomy. Intraoperative view after completion of lymphadenectomy showing clear exposure of the ICA, ICV, MCA, common arterial trunk, and SMV. ICA, ileocolic artery; ICV, ileocolic vein; MCA, middle colic artery; SMV, superior mesenteric vein

Lymphadenectomy was initiated around the SMV, and careful dissection was continued peripherally along the common arterial trunk. After precise identification of the bifurcation between the MCA and ICA, only the ICA was divided, preserving the MCA (**Fig. 3**). Dissection around the complex vascular anatomy was performed using the double bipolar method, which allowed stable and controlled tissue handling while minimizing the risk of vascular injury.

Reconstruction was achieved by intracorporeal overlap anastomosis. Indocyanine green fluorescence imaging was used to confirm adequate perfusion of the bowel stump before intracorporeal anastomosis. The operative time was 383 min, with a console time of 295 min, and the estimated blood loss was 150 mL. Although the patient had no history of previous laparotomy, dense adhesions between the ileocecal region and the abdominal wall required approximately 30 min of careful adhesiolysis. In addition, the patient had a BMI of 24 with relatively abundant visceral fat, which increased the technical difficulty of lymphadenectomy. Furthermore, meticulous dissection around the anomalous common arterial trunk was necessary to ensure oncological safety. These combined factors contributed to the prolonged operative time. The majority of blood loss was due to oozing during adhesiolysis, and no major vascular injury occurred. No transfusion was required. The postoperative course was uneventful, and the patient was discharged without complication. The surgical procedure is demonstrated in **Supplementary Video 1**.

## DISCUSSION

This case provides an important educational message regarding the identification and safe management of rare mesenteric vascular variations during robot-assisted colorectal surgery. Although similar vascular anomalies have been previously reported, the educational novelty of the present case lies in the detailed surgical strategy and technical considerations enabled by precise preoperative CTA and robotic articulation. Anatomical variations of the mesenteric vessels are clinically important because they can significantly increase the technical difficulty and risk of vascular injury during right-sided colon surgery. In particular, variations involving the MCA and ICA may complicate lymphadenectomy around the SMV, especially in minimally invasive procedures where tactile feedback is limited.

Several anatomical and radiological studies have demonstrated considerable variability in the branching patterns of the superior mesenteric artery. Gamo et al. proposed a comprehensive classification based on cadaveric and CT analyses and reported that a common trunk involving the MCA and ICA is extremely rare, accounting for approximately 0.35% of cases on radiological evaluation.^1)^ This rarity highlights the exceptional nature of the present case. Furthermore, Goyo et al. reported a case of ascending colon cancer with a common trunk of the MCA and ICA and emphasized that failure to recognize such vascular anomalies preoperatively may lead to unexpected bleeding or inadequate lymphadenectomy.^2)^ Additionally, incorrect vascular ligation may cause blood flow insufficiency in the remaining bowel, potentially necessitating excessive bowel resection.

In the present case, detailed preoperative contrast-enhanced CT enabled accurate identification of the anomalous arterial anatomy, allowing appropriate surgical planning. Unlike standard right hemicolectomy, confirmation of the bifurcation within the common arterial trunk was essential before vascular division to avoid inadvertent ligation of the MCA. From an oncological perspective, D3 lymphadenectomy was performed according to standard principles for right-sided colon cancer, including dissection of central nodes along the SMV. Selective division of the ICA after confirmation of the bifurcation allowed preservation of the MCA without compromising the extent of lymph node dissection. Robot-assisted surgery provided stable 3D visualization and enhanced instrument articulation, which facilitated precise dissection around the common arterial trunk. Despite extensive adhesions between the ileocecal region and the abdominal wall and a prolonged operative time, lymphadenectomy was safely completed without major bleeding or vascular injury.

In addition to accurate anatomical assessment and the advantages of robot-assisted surgery, the selection of appropriate energy devices played an important role in ensuring procedural safety. The double bipolar method was particularly useful for controlled dissection in a narrow operative field adjacent to major vessels. Compared with monopolar devices, bipolar energy allows precise coagulation with limited thermal spread, which is advantageous when operating near critical vascular structures.^5,6)^ Fujimoto et al. reported on 100 consecutive robot-assisted colorectal cancer resections using the double-bipolar method, demonstrating minimal blood loss, low complication rates, and a manageable learning curve, supporting the feasibility and safety of this approach.^7)^ In this case, the double bipolar method enabled stable tissue handling and effective hemostasis during dissection around the common arterial trunk, even in the presence of severe adhesions, and may have contributed to the absence of high-risk bleeding events.

Taken together, this case demonstrates that thorough preoperative vascular evaluation, combined with robot-assisted surgery and appropriate energy devices, can enable safe management of rare and complex mesenteric vascular variations. Accumulation of similar cases may help establish optimal surgical strategies for right-sided colon cancer with uncommon vascular anatomy.

## CONCLUSIONS

Robot-assisted ileocecal resection can be safely performed in patients with rare vascular variations such as a common trunk of the MCA and ICA when detailed preoperative imaging and meticulous surgical techniques are employed. The use of the double bipolar method may further enhance safety during complex vascular dissection in right-sided colon surgery.

## SUPPLEMENTARY MATERIALS

Supplementary video 1 Robot-assisted ileocecal resection demonstrating identification of a common arterial trunk formed by the middle colic artery and ileocolic artery, and meticulous dissection around the ileocolic vessels using the double bipolar method.

## DECLARATIONS

### Use of artificial intelligence (AI) tools

The authors used an artificial intelligence–assisted language model to improve the clarity and readability of the manuscript. The AI tool was not used to generate scientific content, interpret data, or make clinical decisions. The authors take full responsibility for the content of this manuscript.

### Availability of data and material

The data supporting the findings of this case report are available from the corresponding author upon reasonable request.
